# Understanding what drives efficacious CBT-I interventions: insights from a circadian-enriched digital CBT-I trial in university students

**DOI:** 10.1093/sleep/zsaf397

**Published:** 2025-12-18

**Authors:** Amelia J Scott

**Affiliations:** eCentreClinic, School of Psychological Sciences, Macquarie University, Sydney, Australia

Insomnia disorder affects approximately 20% of university students [1, 2]. The transition into university represents a period of developmental and psychosocial change that heightens vulnerability to insomnia and mental health conditions broadly [3]. Understanding how insomnia develops and is maintained in this population, and ensuring access to effective interventions, is an important clinical and public health priority.

Pape *et al.* [4] emphasize that university life is also characterized by a high likelihood of circadian misalignment [5]. Irregular schedules, evening socializing, shift work, and academic pressures may predispose students to circadian misalignment, and thereby contribute to insomnia onset or maintenance. Circadian processes may therefore represent an important treatment target.

Decades of research supports the efficacy of cognitive behavioral therapy for insomnia (CBT-I) [6, 7], including digital versions [8, 9]. Yet, we still know little about which CBT-I components are most important, and for whom. Mechanistic trials are needed to move the field from “does CBT-I work?” to “what within CBT-I works, for whom, and why?”

This study addresses one such question: does enriching CBT-I with circadian-specific content offer added value for university students? The authors examined this through a pragmatic randomized controlled trial (RCT) comparing a guided CBT-I program enriched with circadian components to an active psychoeducation and monitoring control condition.

Participants (*n* = 195) were randomized to receive guided digital CBT-I with circadian enrichment (“i-Sleep & BioClock”), or a less intensive control program with brief psychoeducation and no clinical guidance. The intervention showed good acceptability and safety. However, the authors did not detect evidence of a treatment effect, with non-significant group-by-time interactions across most primary and secondary outcomes. Dropout was substantial (53% in treatment, 43% in control), which, although anticipated and not unusual in digital interventions and university populations [9, 10], warrants consideration.

Notable strengths of the study include its pragmatic design, minimal exclusion criteria, and delivery in a real-world university setting. Digital delivery also increases accessibility for a population that struggles to engage with traditional in-person services. The use of an active control condition provides a more stringent test than passive comparators (e.g. waitlist, no-treatment). Enriching the intervention with circadian content aligns with emerging evidence for the importance of circadian processes in youth sleep and mental health.

The null findings of the study should not be interpreted as evidence that digital CBT-I (with or without circadian enrichment) “is not effective” for university students. Several statistical, sample-, and design-related considerations help contextualize the findings.

At the primary endpoint, large within-group improvements were observed; d = 1.72 (6.6 points on the insomnia severity index (ISI)) for the intervention and d = 1.42 (5.4 ISI points) for the control. Although the between-group difference (d = 0.31) was not statistically significant, it sits within a range typically be considered clinically relevant in trials comparing two active psychological interventions. For example, a review of placebo-controlled randomized trials across medicine and psychiatry reported median effect sizes of 0.37 and 0.41, respectively [11]. Incremental effects in such designs are typically small and require large samples to detect reliably.

The high dropout further reduces precision; only 101 participants (52%) contributing data at the primary endpoint. Although the study was powered for moderate effects and expected this much attrition, substantial dropout can still reduce precision in ways that are difficult to anticipate. Attrition introduces uncertainty because the outcomes of those who disengage are unknown and may not resemble the trajectories of those who remain. The authors’ pattern-mixture sensitivity analyses suggested no major departures from the missing-at-random assumption, yet it remains possible that participants who dropped out followed different outcome patterns. These considerations reduce statistical power and widen the uncertainty around the observed effects.

Taken together, the null finding may therefore reflect insufficient sensitivity to detect modest but meaningful additive effects of circadian enrichment, rather than evidence that such effects are absent.

The use of an active control condition also helps us understand the current results. Without a passive or waitlist comparator, it is difficult to estimate the degree of improvement attributable to the passage of time, expectancy, or non-specific therapeutic factors. In waitlist controls, ISI reductions typically fall in the range of 2-3 points [12, 13], which is smaller than the 5.4-point reduction observed in this study. This may suggest that a brief, lower-dose intervention was sufficient for some participants, particularly given their educational background and familiarity with online learning. This is consistent with emerging evidence that very brief digital interventions can be equivalent in efficacy to standard-length counterparts [14]. Higher module completion in the control arm also suggests that brevity and simplicity may have supported participants’ engagement.

It is also helpful to consider the well-documented progression from acute to chronic insomnia. Approximately 70% of adults who experience acute insomnia will remit without treatment within several weeks or months [15]. Among remitters, the primary mechanism driving improvement appears to be *time.* Chronic insomnia, however, is characterized not by the initial trigger but by the emergence of perpetuating mechanisms, such as heightened sleep-related anxiety, conditioned arousal, excessive time in bed, or circadian misalignment.

Given that eligibility for this trial was based on ISI ≥10 without a chronicity requirement, a sizeable proportion of participants may have been experiencing situational or stress-related acute insomnia. For these individuals, symptoms may have improved spontaneously, and this may have contributed to the high dropout rate and reduced engagement during the study period. In this sense, the pragmatic strengths of the study may have come at a cost of a more precise investigation of treatment effects and of the mechanisms that perpetuate insomnia in this population.

To evaluate whether circadian enrichment of CBT-I provides added value for university students, it is crucial that the enriched component is cleanly isolated. In this trial, both arms included some circadian information, albeit with differences in depth and emphasis. The treatment and control groups also differed in the number and length of modules, as well as in the presence of guided support. These design features make it difficult to determine whether any between-group reflect circadian enrichment specifically, the effect of clinical guidance, or simply a higher dose of treatment.

A more definitive mechanistic test would involve two structurally equivalent conditions with the same degree of guidance and differing only in the presence versus absence of circadian enrichment (with the control arm receiving a matched “distractor” module of equivalent length and intensity). Given the multifactorial nature of insomnia, any incremental effect of circadian content is likely to be modest, yet potentially meaningful at both theoretical and clinical levels. Adequate powering is therefore essential. Such a design would offer clearer evidence regarding whether circadian content confers incremental benefit and, indirectly, whether circadian misalignment meaningfully perpetuates insomnia in university students.

To conclude, Pape and colleagues [4] have contributed a pragmatic and thoughtfully-designed study that addresses a clinically meaningful question in a population with clear need. Yet some of the features that increase its real-world relevance (minimal exclusion criteria, digital delivery, and an active comparator) also make it more challenging to draw firm conclusions. Even so, the findings offer useful guidance for future research. There is a clear opportunity to determine the optimal treatment dose for university students, to test whether circadian enrichment has greater impact in those with more chronic presentations, and to conduct more tightly controlled dismantling trials with adequate power. Progress here will deepen our understanding of the mechanisms that perpetuate insomnia in young adults, and inform the development of interventions that best meet their needs.

## References

[1] Sivertsen B, Vedaa Ø, Harvey AG, et al. Sleep patterns and insomnia in young adults: a national survey of Norwegian university students. *J Sleep Res*. 2019;28(2):e12790. 10.1111/jsr.12790 30515935

[2] Jiang X-L, Zheng X-Y, Yang J, et al. A systematic review of studies on the prevalence of insomnia in university students. *Public Health*. 2015;129(12):1579–1584. 10.1016/j.puhe.2015.07.030 26298588

[3] Sharp J, Theiler S. A review of psychological distress among university students: pervasiveness, implications and potential points of intervention. *Int J Adv Counsel*. 2018;40(3):193–212. 10.1007/s10447-018-9321-7

[4] Pape L, van Straten A, Struijs S, Karch J, Spinhoven P, Antypa N. Effects of a guided digital intervention on sleep and mental health outcomes in university students - a randomized controlled trial. *Sleep*. 2026;49(5):zsaf357. 10.1093/sleep/zsaf357 PMC13163168 41288591

[5] Yeom JW, Park S, Lee H-J. Managing circadian rhythms: a key to enhancing mental health in college students. *Psychiatry Investig*. 2024;21(12):1309. 10.30773/pi.2024.0250 PMC11704804 39757810

[6] Furukawa Y, Sakata M, Yamamoto R, et al. Components and delivery formats of cognitive behavioral therapy for chronic insomnia in adults: a systematic review and component network meta-analysis. *JAMA Psychiatry*. 2024;81(4):357–365. 10.1001/jamapsychiatry.2023.5060 38231522 PMC10794978

[7] Trauer JM, Qian MY, Doyle JS, Rajaratnam SM, Cunnington D. Cognitive behavioral therapy for chronic insomnia: a systematic review and meta-analysis. *Ann Intern Med*. 2015;163(3):191–204. 10.7326/M14-2841 26054060

[8] Soh HL, Ho RC, Ho CS, Tam WW. Efficacy of digital cognitive behavioural therapy for insomnia: a meta-analysis of randomised controlled trials. *Sleep Med*. 2020;75:315–325. 10.1016/j.sleep.2020.08.020 32950013

[9] Zachariae R, Lyby MS, Ritterband LM, O'Toole MS. Efficacy of internet-delivered cognitive-behavioral therapy for insomnia–a systematic review and meta-analysis of randomized controlled trials. *Sleep Med Rev*. 2016;30:1–10. 10.1016/j.smrv.2015.10.004 26615572

[10] Dear BF, Johnson B, Singh A, et al. Examining an internet-delivered intervention for anxiety and depression when delivered as a part of routine care for university students: a phase IV trial. *J Affect Disord*. 2019;256:567–577. 10.1016/j.jad.2019.06.044 31280082

[11] Leucht S, Hierl S, Kissling W, Dold M, Davis JM. Putting the efficacy of psychiatric and general medicine medication into perspective: review of meta-analyses. *Br J Psychiatry*. 2012;200(2):97–106. 10.1192/bjp.bp.111.096594 22297588

[12] Lancee J, van Straten A, Morina N, Kaldo V, Kamphuis JH. Guided online or face-to-face cognitive behavioral treatment for insomnia: a randomized wait-list controlled trial. *Sleep.* 2016;39(1):183–191. 10.5665/sleep.5344 26414893 PMC4678352

[13] Horsch CH, Lancee J, Griffioen-Both F, et al. Mobile phone-delivered cognitive behavioral therapy for insomnia: a randomized waitlist controlled trial. *J Med Internet Res*. 2017;19(4):e70. 10.2196/jmir.6524 28400355 PMC5405291

[14] Bisby MA, Balakumar T, Scott AJ, Titov N, Dear BF. An online therapist-guided ultra-brief treatment for depression and anxiety: a randomized controlled trial. *Psychol Med*. 2024;54(5):902–913. 10.1017/S003329172300260X 37655527

[15] Perlis ML, Vargas I, Ellis JG, et al. The natural history of insomnia: the incidence of acute insomnia and subsequent progression to chronic insomnia or recovery in good sleeper subjects. *Sleep.* 2020;43(6). 10.1093/sleep/zsz299 PMC7294401 31848629

